# Mimicking B and T cell epitopes between *Mycobacterium leprae* and host as predictive biomarkers in type 1 reaction in leprosy

**DOI:** 10.1038/s41598-021-04135-5

**Published:** 2021-12-24

**Authors:** Vinay Kumar Pathak, Itu Singh, Shoor Vir Singh, Utpal Sengupta

**Affiliations:** 1 Stanley Browne Laboratory, The Leprosy Mission Community Hospital, Nand Nagari, Shahdara, New Delhi, India 110093; 2 GLA University, Mathura, UP India 281406

**Keywords:** Immunology, Biomarkers, Diseases, Medical research

## Abstract

Several Mycobacterial infections including leprosy and tuberculosis are known to evoke autoimmune responses by modulating homeostatic mechanism of the host. Presence of autoantibodies like, rheumatoid factor, anti-nuclear factor and antibodies to host, collagen, keratin, myelin basic protein (MBP) and myosin, have been earlier reported in leprosy patients. In the present study, we detected the role of mimicking epitopes between *Mycobacterium leprae* and host components in the induction of autoimmune response in leprosy. Based on our previous findings, we predicted and synthesized a total of 15 mimicking linear B cell epitopes (BCE) and 9 mimicking linear T cell epitopes (TCE) of keratin and MBP. Humoral and cell-mediated immune responses against these epitopes were investigated in Non-reaction (NR), Type 1 reaction (T1R) leprosy patients, and healthy controls. We observed significantly higher levels of antibodies against 8 BCE in T1R in comparison to NR leprosy patients. Further, we also found 5 TCE significantly associated with lymphocyte proliferation in the T1R group. Our results indicated that these epitopes play a key role in the induction of autoimmune response in leprosy and are also strongly associated with the inflammatory episodes of T1R. Conclusively, these molecules may be employed as a biomarker to predict the inflammatory episodes of T1R.

## Introduction

Biotic factors such as bacteria of the ecosystem might induce an imbalance in the homeostatic mechanism of the host leading to an autoimmune response. Mycobacteria have been found to be able to modulate host immune responses both in humans and experimental animals^1^.

Leprosy is a chronic granulomatous disease caused by *Mycobacterium leprae*, affecting mainly peripheral nerves, skin, and mucous membrane. Reactions in leprosy are immunological complicated state that occurs either before, during, or after treatment and affect 30–50% of patients with leprosy^2, 3^. Type 1 reaction (T1R) is characterized by acute inflammation of skin lesions and/or nerves and is a major cause of neuritis often leading to nerve damage resulting in deformities of mainly hands and feet. The immune response is characteristic of delayed type hypersensitivity (DTH) reaction with an influx of peripheral blood lymphocytes demonstrating an increased reactivity to *M. leprae* antigens in a lymphocyte transformation test (LTT)^4^. This may lead to a local decrease in bacillary load and augmentation of T cell reactivity in the affected nerve leading to nerve damage^5^.

Antigenic/molecular mimicry between host and pathogen has been suggested as a way to escape detection and destruction of the pathogen by host immunity^6^. Antigenic mimicry might be one of the reasons for escaping the immune surveillance of *M. leprae* and its long period of incubation before the onset of the disease. The resemblance of epitopes may facilitate the survival of *M. leprae* in the human hosts when the antigens are not recognized as “non-self”, a situation that seems to occur in lepromatous leprosy when the patient’s tissues are loaded with bacteria virtually without any protective immune response. On the other hand, *M. leprae* antigens which mimic host antigens may induce an autoimmune reaction against the host’s own antigens. The antigenic molecular mimicry between pathogen and host may play a role in the pathogenesis of leprosy as well as it may be one of the reasons for immunological complications. Earlier, highest level of anti-keratin, anti-MBP and anti-myosin antibodies has been reported in T1R group of leprosy^7–9^. However, mimicking epitopes responsible for immunogenicity has not been studied extensively in leprosy. Hence, the present study was carried out to understand the immunological response to mimicking B and T cell epitopes across two different clinical spectra of leprosy and to elucidate their role in the induction of autoimmune response in T1R group of leprosy.

Our main research question was to evaluate whether autoantibodies in the T1R group of leprosy produced because of mimicking epitopes of host protein/s and *M. leprae* protein/s. Hence, the major objectives of this study were, firstly to evaluate antibody level against mimicking B cell epitopes across two groups, i.e., T1R and borderline leprosy patients without reaction (NR) for their humoral immune response to these mimicking epitopes and secondly to evaluate lymphocyte proliferation activity against the mimicking T cell epitopes in these groups to probe into their state of cell-mediated immune response. The healthy group was taken as the control variable group.

## Material and methods

### Recruitment of subjects

This work was carried out after getting approval from the Institutional Ethics Committee (IEC) of The Leprosy Mission Trust India, New Delhi (Dated June 24, 2013). Blood samples were obtained from leprosy patients and healthy controls after obtaining written informed consent at the outpatient department at The Leprosy Mission Community Hospital, Delhi. Sample collection protocols and all methods in the study were performed with strict adherence to the guidelines and regulations of IEC, The Leprosy Mission Trust India (TLMTI), New Delhi and the Indian Council of Medical Research (ICMR), New Delhi.

#### Patients and healthy controls

A total of 100 clinically diagnosed cases (based on cardinal signs and bacteriological examination) consisting of 50 untreated leprosy patients in T1R and 50 untreated NR were recruited for the study. The inclusion criteria for patients were leprosy patients (NR and T1R) between ages of 18 years and 60 years, the patients below the age of 18 years and above 60 years were not included. Leprosy patients co-infected with other infections like dermatological infections, TB, HIV, Immune disorders, autoimmune diseases, and pregnant women were excluded from the study. Twenty-two non-occupational healthy controls without any clinical history of leprosy, TB, and other dermatologic infections and other diseases were included in the study from non-endemic areas. Lymphocyte proliferation assay was performed with 10 healthy controls, 20 untreated NR, and 20 untreated T1R patients. The demographic characteristics and clinical data of patients are given as below (Table 1).

**Table 1:** Demographic characteristics of leprosy patients and healthy controls.

Characteristics	Non-reaction (n 50)	Type 1 reaction (n 50)	Healthy controls (n 22)
Age (mean ± SD)	35.18 ± 15.28	39 ± 13.14	32.69 ± 10.95
**Gender (%)**			
Male	31 (62%)	34 (68%)	15 (68.18%)
Female	19 (38%)	16 (32%)	7 (31.81%)
Bacillary index (mean ± SD)	1.65 ± 1.15	2.34 ± 1.41	–
**Duration of MDT**			
0 month	0	12	–
1–2 months	0	8	–
2–3 months	0	17	–
3–4 months	0	13	–

### Sample collection

Forth the first objective, blood samples (≈ 5 ml each) were aseptically collected from all the subjects. Sera were separated and stored at − 20 °C till these were further used for antibody estimation against mimicking epitopes and for the second objective blood samples (≈ 8 ml each) were aseptically collected from all the subjects and processed further for PBMC isolation and then the 3-(4,5-dimethylthiazol-2-yl)-2,5-diphenyl -tetrazolium bromide (MTT) assay was performed against mimicking T cell epitopes.

### Synthesis of mimicking epitopes

#### B cell epitopes

Mimicking B cell epitopes. of *M. leprae* predicted by BCPREDS server 1.0 and host protein/s identified by ClustalW multiple sequence alignment server were identified and the linear peptide epitopes/ were synthesized commercially by Thermo-Scientific, USA. A total of eleven B cell mimicking epitopes of protein HSP65 (*M. leprae*) and protein keratin (host) as well as four B cell mimicking epitopes of protein 50S ribosomal protein, lysyl tRNA synthetase (*M. leprae*), and myelin basic protein (host) were synthesized. In brief, The B cell epitopes/linear peptides synthesized included seven mimicking B cell epitopes of HSP65 of *M. leprae* (similar with the keratin of host) namely HSP1, HSP2, HSP3, HSP4, HSP5, HSP6, and HSP7; four mimicking B cell epitopes of keratin (similar with HSP65 of *M. leprae*), i.e. KER1, KER2, KER3, and KER4; two mimicking B cell epitopes of myelin basic protein (similar to 50S ribosomal protein of *M. leprae*) MBP50SB1 and MBP50SB2; two mimicking B cell epitopes of myelin basic protein (similar to lysyl tRNA synthetase of *M. leprae*) MBPLMB1 and MBPLMB2 were synthesized. All the peptides/ epitopes synthesized were of > 98% HPLC grade purity.

#### T cell epitopes

T cell epitopes were predicted by Syfpeithi epitope prediction server and among them mimicking T cell epitopes of *M. leprae* and host protein/s were identified by ClustalW multiple sequence alignment server. A total of nine T cell mimicking epitopes/linear peptides of protein 50S ribosomal protein and Lysyl tRNA synthetase of *M. leprae* similar to myelin basic protein (host) were predicted. Similar to B cell epitopes, the T cell epitopes/linear peptides were also synthesized commercially by Thermo-Scientific, USA. In brief, The T cell epitopes/linear peptides synthesized included two mimicking T cell epitopes of 50S ribosomal protein of *M. leprae* (similar with MBP), i.e., 50ST1, 50ST2; two mimicking T cell epitopes of Lysyl tRNA synthetase of *M. leprae* (similar to MBP) i.e. LMT1, LMT2; three mimicking T cell epitopes of MBP (similar with 50S ribosomal protein of *M. leprae*), i.e., MBP50ST1, MBP50ST2, MBP50ST3 and two mimicking T cell epitopes of MBP (similar to lysyl tRNA synthetase of *M. leprae*), i.e., MBPLMT1 and MBPLMT2.

### Standardization

Standardization of ELISA with B cell epitopes and MTT assay with T cell epitopes was performed by using checkerboard analysis and we found that 2.5 µg/ml concentration of predicted B cell epitopes/peptides and 1:1600 ratio dilution of serum sample were most suitable parameters to perform the test. Similarly, it was observed that 10 µg/ml concentration of T cell epitopes was the appropriate dose for MTT assay.

Thereafter, ELISA was performed to evaluate humoral immune response against mimicking B cell epitopes across groups i.e., T1R, NR and healthy control. In brief, 96 well Microtiter plates were coated with mimicking B cell epitopes/peptides (Conc. 2.5 µg/ml) dissolved in 0.05 M Carbonate—bicarbonate buffer (pH 9.6) and incubated overnight at 37 °C. Thereafter, the plates were washed with PBS (pH 7.4), then blocked with 2% BSA in PBS and incubated at 37 °C for 1 h. Followed by washing with PBS the diluted serum samples were added [Dilution factor 1:1600 in 1% BSA in PBS-Tween 20 (0.05%)] and plates were incubated at 37 °C for 2 h. Later on, the washing was done with PBS -Tween 20 (0.05%) [PBST], then Anti-human IgG peroxidase conjugate (Cat. No. A8792, Sigma Aldrich, USA) diluted in 1% BSA in PBS-Tween 20 (0.05%) was added and plates were incubated at 37 °C for 1 h. Afterward, plates were washed with PBST, and substrate solution of O-Phenyldiamine (Conc. 0.5 mg/ml) was added with 0.5% H_2_O_2_ and incubated for 20 min in dark at RT. Thereafter, the reactions were stopped with a solution of 2 N H_2_SO_4,_ and OD was determined at 492 nm by using MULTISKAN FC ELISA Reader (Thermo Scientific, USA).

Similarly, lymphocyte proliferation activity against the mimicking T cell epitopes was performed to investigate cell-mediated immune response across the same groups. PBMCs were isolated by the Ficoll-Hypaque density gradient centrifugation method^10^. In brief, PBMCs isolated from leprosy patients and healthy controls were suspended in culture media RPMI—1640 (Sigma, USA) containing 5% of heat-inactivated fetal bovine serum (FCS) (Thermo Fisher Scientific, USA), 2 mM of l-glutamine (Sigma, USA), 2 mM of antibiotic antimycotic solution (Sigma, USA), and a suspension containing 2 × 10^6^ viable cells/ml was made. The cells were dispensed 2 × 10^5^ cells per well in 96 well flat-bottomed plates and stimulation was given by adding 10 µg/ml concentration of T cell epitopes/peptides. The plates were incubated in humidified environment at 37 °C in a CO_2_ incubator (Eppendorf, Germany) at a 5% CO_2_ level, for 72 h. Thereafter, lymphocyte proliferation assay was performed by adding MTT [3-(4,5-dimethylthiazol-2-yl)-2,5-diphenyltetrazolium bromide] solution (5 mg/ml) to each well and incubated for 4 h at 37 °C. The formazan crystals thus formed were dissolved with 100 µl of lysis buffer (Equal volume of DMSO and absolute ethanol mixed with 0.0004% SDS). After a few minutes of incubation at RT, the OD was determined at 540 nm by using MULTISKAN FC ELISA Reader (Thermo Scientific, USA). The stimulation index was calculated by using the formula: Stimulation index = A 540 nm with stimulant/A 540 nm without stimulant, where A 540 = Absorbance at 540 nm.

### Ethics approval and consent to participate

The study was approved by the Institutional Ethics Committee (IEC) of The Leprosy Mission Trust India, New Delhi (Dated June 24, 2013). The IEC of The Leprosy Mission Trust India is associated with the The Leprosy Mission Community Hospital, Delhi. All participants were recruited after obtaining written informed consents at the outpatient department.

## Results

**Figure 1:**
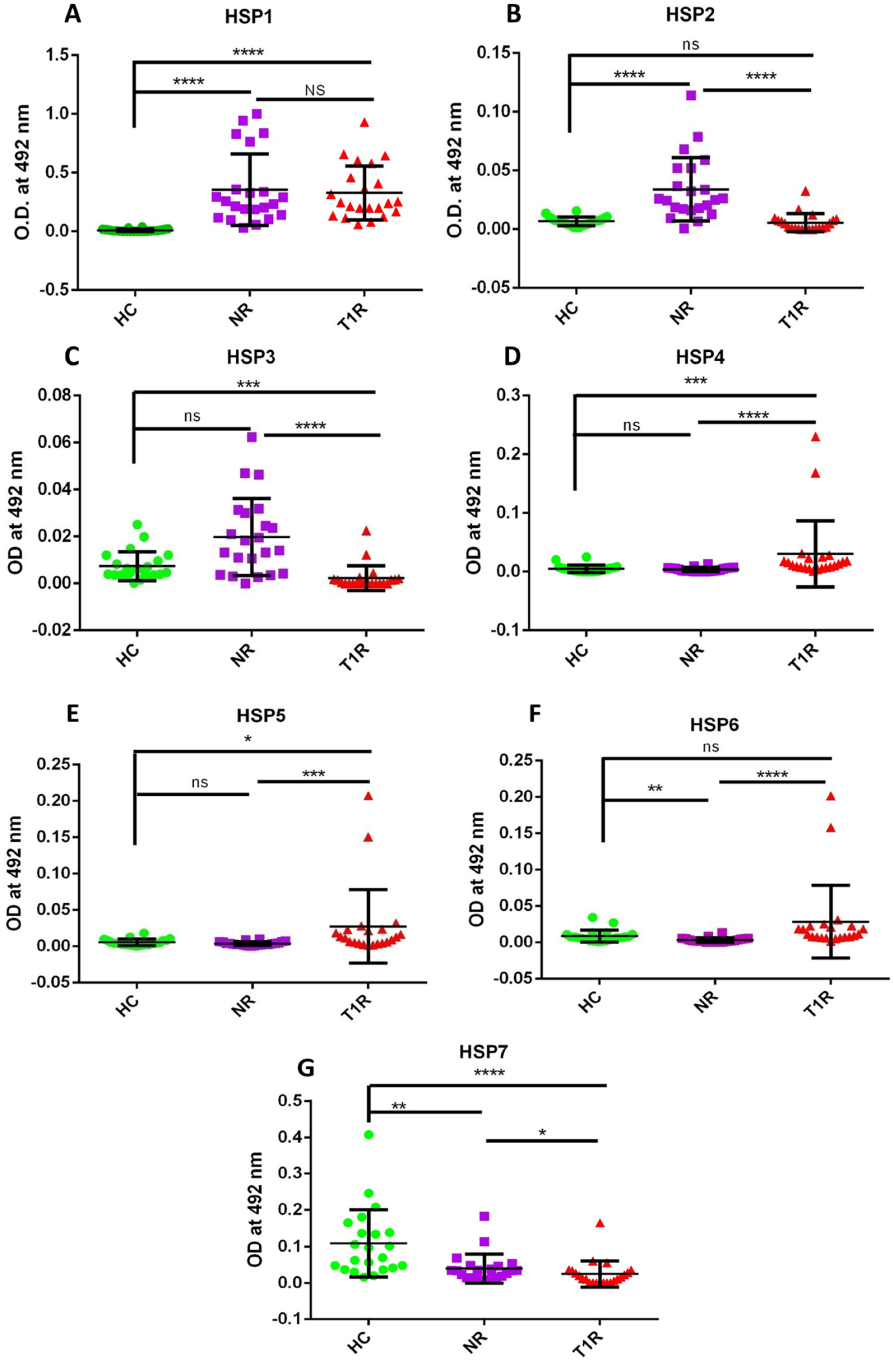
Level of antibodies against **7** HSP65 of *M. leprae* peptides (mimicking B cell epitopes) in the sera of leprosy patients and healthy controls, (**A**) HSP1, (**B**) HSP2, (**C**) HSP3, (**D**) HSP4, (**E**) HSP5, (**F**) HSP6, (**G**) HSP7. Smooth vertical lines along with horizontal lines represent the mean OD value with SD of each group. Each dot represents the OD value at 492 nm of each individual. One-way ANOVA (and non-parametric test) and multiple comparison test (Dunn’s test) were used to find out the difference between OD value obtained in the sera of healthy controls and leprosy patients. (****p value < 0.0001, ***p value < 0.001, **p value < 0.01, *p value < 0.05, ns = not significant).

The humoral immune response against each mimicking B cell epitope of keratin and myelin basic protein was estimated by the level of auto antibodies. Antibodies against HSP1, HSP2, HSP3 were found to be higher in NR when compared to T1R (ns, p < 0.0001 and p < 0.0001 respectively); however, the mean level of antibodies against HSP1 was found to be significantly higher in both T1R and NR when compared to HC (p < 0.0001). Among all mimicking HSP65 peptides screened, it was observed that the mean level of antibodies against HSP4 and HSP5 were significantly higher in T1R when compared to NR (p < 0.0001, p < 0.001, respectively) and HC (p < 0.001, p < 0.05, respectively). The mean level of antibodies against HSP6 was higher in T1R when compared to NR (p < 0.0001) and HC (p = ns). The antibody levels against HSP7 were found to be higher in HC followed by NR (p < 0.01) and T1R (p < 0.0001). (Table 2, Fig. 1).

**Table 2:** Mean OD values in leprosy patients and healthy controls against each mimicking B cell epitopes of HSP65 of *M. leprae.*

Sr. no.	Peptide	Healthy controls (n = 22)	Non-reaction (n = 50)	Type 1 reaction (n = 50)
1	HSP1 (mean ± SD)	0.0077 ± 0.012	0.355 ± 0.30	0.327 ± 0.23
2	HSP2 (mean ± SD)	0.0067 ± 0.004	0.0340 ± 0.027	0.0055 ± 0.008
3	HSP3 (mean ± SD)	0.0074 ± 0.006	0.0198 ± 0.016	0.0023 ± 0.005
4	HSP4 (mean ± SD)	0.0050 ± 0.006	0.0038 ± 0.003	0.030 ± 0.054
5	HSP5 (mean ± SD)	0.0055 ± 0.004	0.0038 ± 0.003	0.027 ± 0.049
6	HSP6 (mean ± SD)	0.0086 ± 0.008	0.0031 ± 0.003	0.028 ± 0.049
7	HSP7 (mean ± SD)	0.108 ± 0.091	0.0392 ± 0.003	0.022 ± 0.037

**Figure 2:**
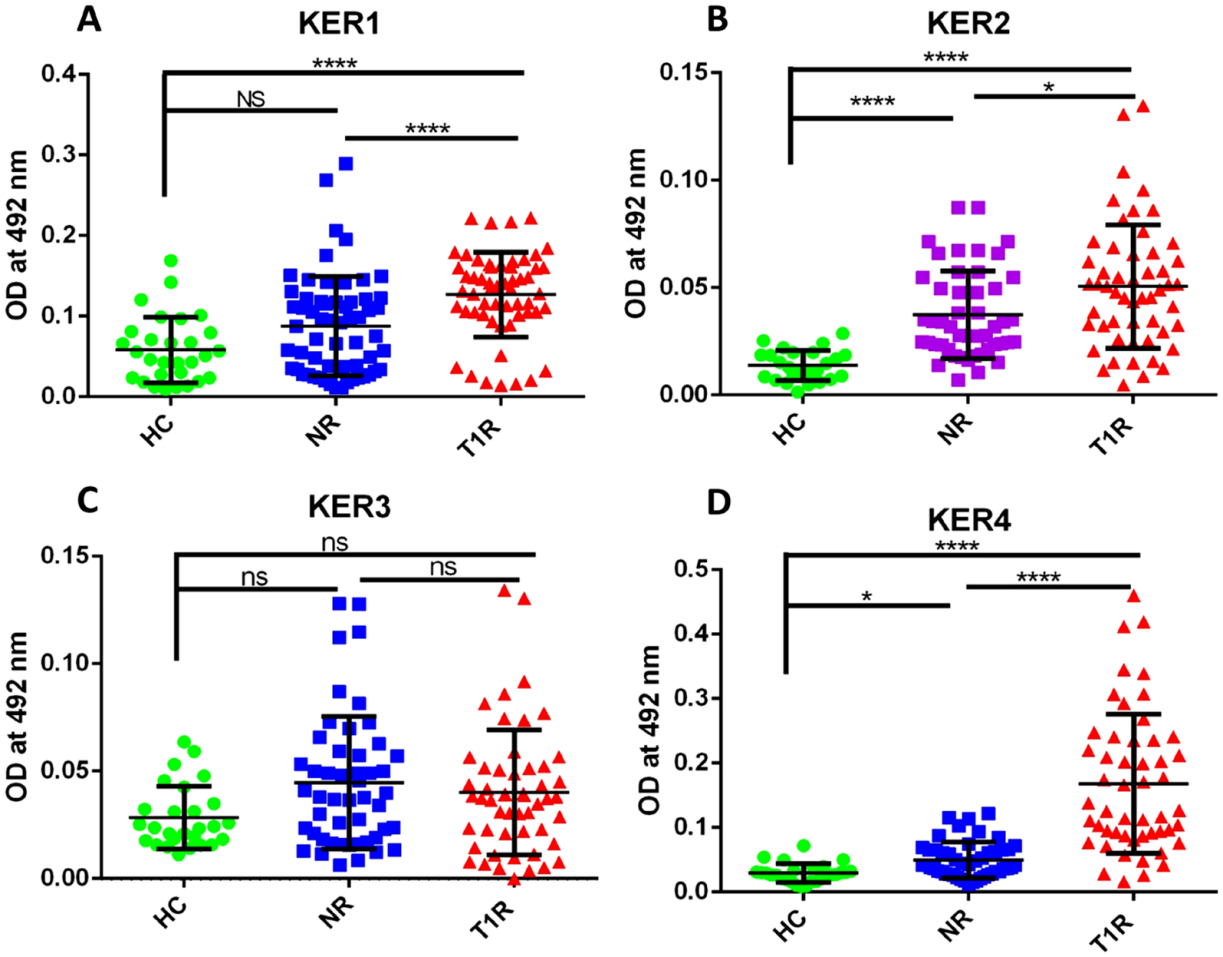
Level of antibodies against 4 Keratin peptides (mimicking B cell epitopes) in the sera of leprosy patients and healthy controls, (**A**) KER1, (**B**) KER2, (**C**) KER3, (**D**) KER4. Smooth vertical lines along with horizontal lines represent the mean OD value with SD of each group. Each dot represents the OD value at 492 nm of each individual. One-way ANOVA (and non-parametric test) and multiple comparison test (Dunn’s test) were used to find out the difference between OD value obtained in the sera of healthy controls and leprosy patients (****p value < 0.0001, ***p value < 0.001, **p value < 0.01, *p value < 0.05, ns = not significant).

We observed significantly higher antibody levels in T1R against mimicking Keratin B cell epitopes i.e. KER1, KER2, KER4 when compared to NR (p < 0.0001, p < 0.05, and p < 0.0001, respectively) and HC (p < 0.0001). Only one peptide i.e. KER3 showed no significant difference between the groups (p > 0.05). (Table 3, Fig. 2).

**Table 3:** Mean OD values in leprosy patients and healthy controls against each mimicking B cell epitopes of keratin of host.

Sr. no.	Peptide	Healthy controls (n = 22)	Non-reaction (n = 50)	Type 1 reaction (n = 50)
1	KER1 (mean ± SD)	0.0582 ± 0.04	0.0877 ± 0.061	0.127 ± 0.052
2	KER2 (mean ± SD)	0.0138 ± 0.007	0.0374 ± 0.02	0.051 ± 0.029
3	KER3 (mean ± SD)	0.0283 ± 0.014	0.045 ± 0.030	0.040 ± 0.029
4	KER4 (mean ± SD)	0.029 ± 0.015	0.050 ± 0.028	0.178 ± 0.1

**Figure 3:**
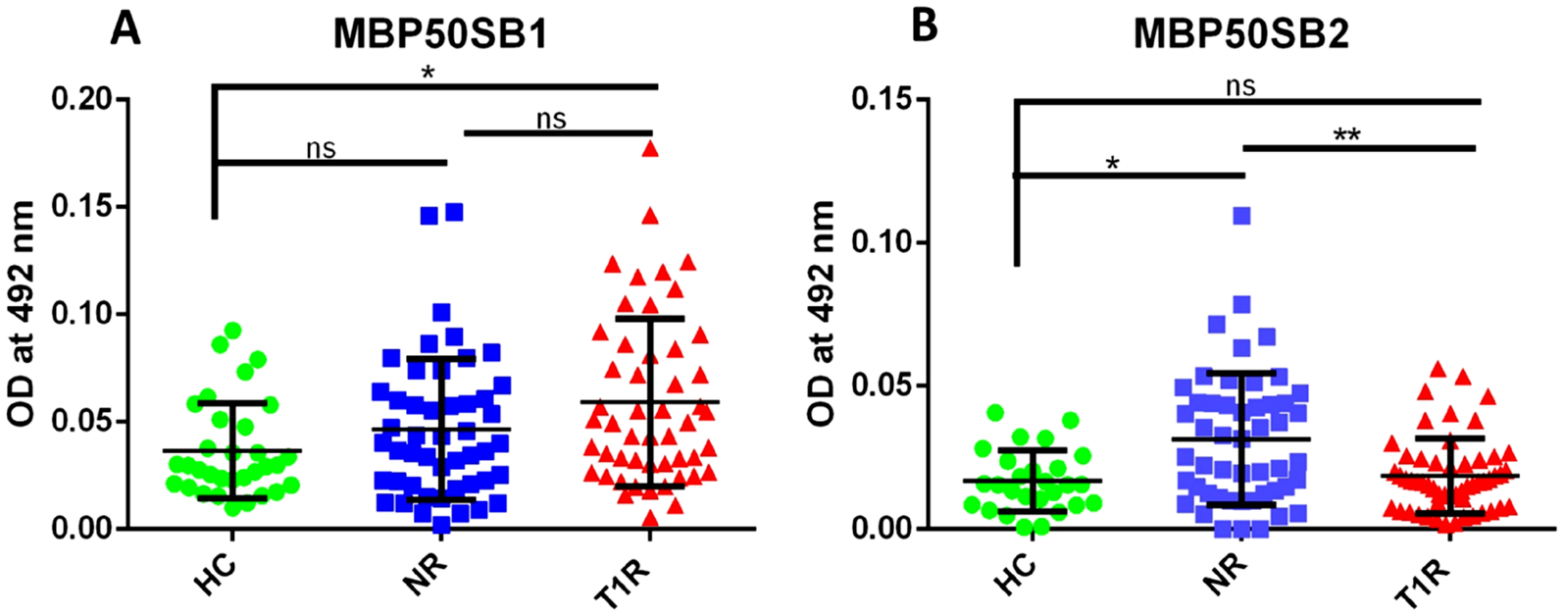
Level of antibodies against two MBP peptides (MBP50S) (mimicking B cell epitopes with 50S ribosomal protein L2) in the sera of leprosy patients and healthy controls, (**A**) MBP50SB1, (**B**) MBP50SB2. Smooth vertical lines along with horizontal lines represent the mean OD value with SD of each group. Each dot represents the OD value at 492 nm of each individual. One-way ANOVA (and non-parametric test) and multiple comparison test (Dunn’s test) were used to find out the difference between OD value obtained in the sera of healthy controls and leprosy patients (**p value < 0.01, *p value < 0.05, ns = not significant).

**Figure 4:**
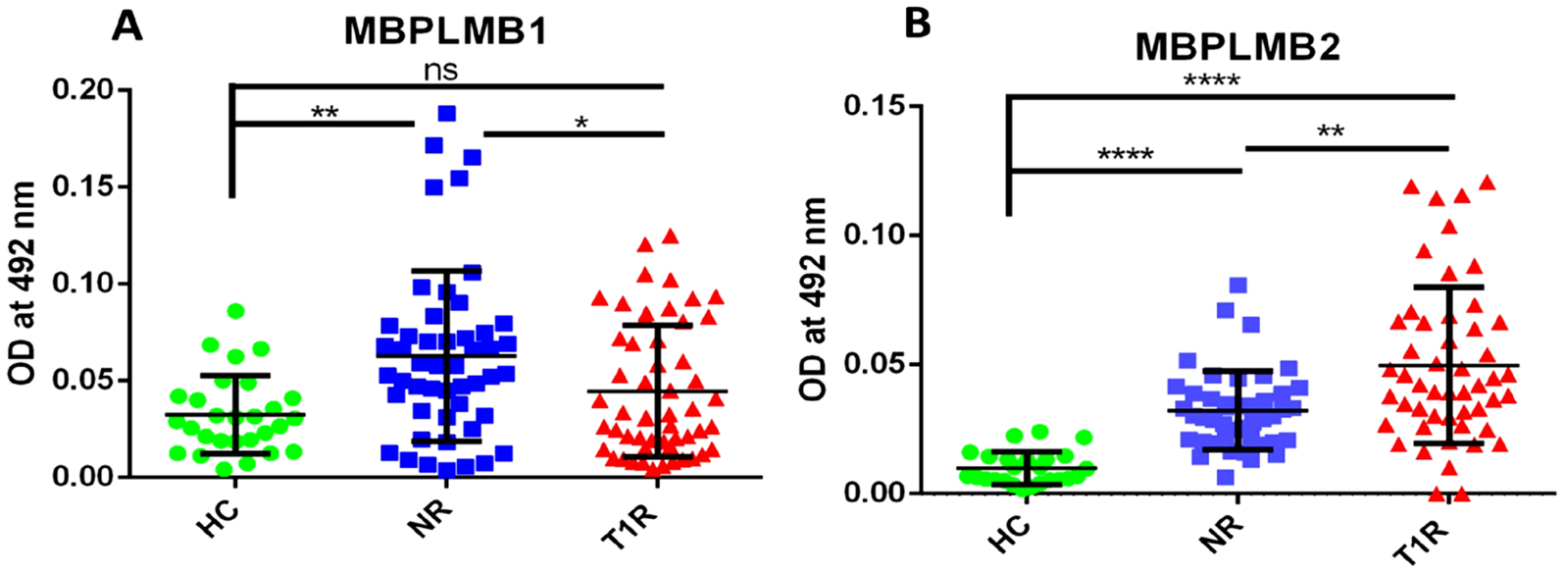
Level of antibodies against two MBP peptides (MBPLM) (mimicking B cell epitopes with Lysyl tRNA synthetase) in the sera of leprosy patients and healthy controls, (**A**) MBPLMB1, (**B**) MBPLMB2. Smooth vertical lines along with horizontal lines represent the mean OD value with SD of each group. Each dot represents the OD value at 492 nm of each individual. One-way ANOVA (and non-parametric test) and multiple comparison test (Dunn’s test) were used to find out the difference between OD value obtained in the sera of healthy controls and leprosy patients (****p value < 0.0001, ***p value < 0.001, **p value < 0.01, *p value < 0.05, ns = not significant).

In the case of two mimicking B cell epitopes of Myelin Basic Protein (similar to 50S ribosomal protein of *M. leprae*) MBP50SB1 and MBP50SB2, we observed significantly higher antibody levels in leprosy patients when compared to HC (p < 0.05 and p = ns, respectively). We also found a higher level of antibodies against MBP50SB1 in T1R and a lower level against MBP50SB2 when compared to NR (ns and p < 0.01, respectively). Similarly, we observed a higher level of antibodies in leprosy patients when compared to HC for two mimicking B cell epitopes of MBP (similar to lysyl tRNA synthetase) MBPLMB1 and MBPLMB2. We also found a significantly lower level of antibodies in T1R for MBPLMB1 (p < 0.05) and a significantly higher level for MBPLMB2 (p < 0.01) when compared to NR, (Table 4, Figs. 3 and 4).

**Table 4:** Mean OD values in leprosy patients and healthy controls against each mimicking B cell epitopes of myelin basic protein.

Sr. no.	Peptide	Healthy controls (n = 22)	Non-reaction (n = 50)	Type 1 reaction (n = 50)
1	MBP50SB1 (mean ± SD)	0.037 ± 0.022	0.047 ± 0.033	0.059 ± 0.039
2	MBP50SB2 (mean ± SD)	0.017 ± 0.010	0.031 ± 0.023	0.019 ± 0.013
3	MBPLMB1 (mean ± SD)	0.032 ± 0.020	0.063 ± 0.044	0.045 ± 0.034
4	MBPLMB2 (mean ± SD)	0.0099 ± 0.006	0.032 ± 0.015	0.049 ± 0.032

### Cell-mediated immune response to mimicking T cell epitopes

We observed that among four mimicking T cell epitopes of 50S ribosomal protein and Lysyl tRNA synthetase of *M. leprae*, significantly higher stimulation index (SI) in the T1R group was found with 50ST2 and LMT1 when compared to NR (p < 0.01 and p < 0.01, respectively) and HC (p < 0.001 and p < 0.01, respectively).

**Figure 5:**
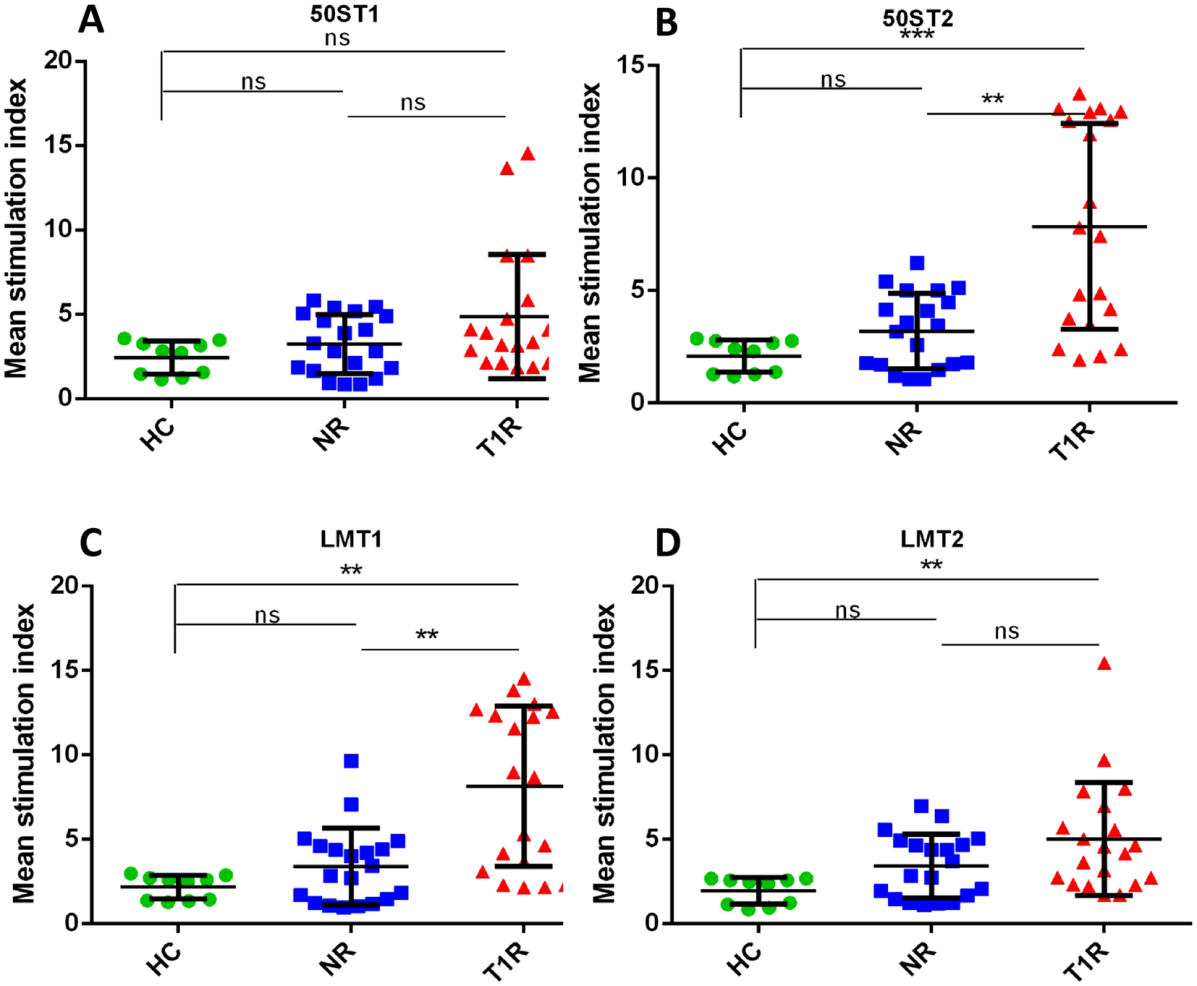
Graph showing mean stimulation index of healthy controls and leprosy patients with mimicking T cell epitopes of 50S ribosomal protein of *M. leprae* (**A**) 50ST1, (**B**) 50ST2 and mimicking T cell epitopes of Lysyl tRNA synthetase of *M. leprae* (**C**) LMT1, (**D**) LMT2. Smooth vertical lines along with horizontal lines represent the mean stimulation index (SI) in each group. Each dot represents the SI of each individual. One-way ANOVA (and non-parametric test) and multiple comparison test (Dunn’s test) were used to find out the difference between SI value obtained in healthy controls and leprosy patients (***p value < 0.001, **p value < 0.01, ns = not significant).

**Figure 6:**
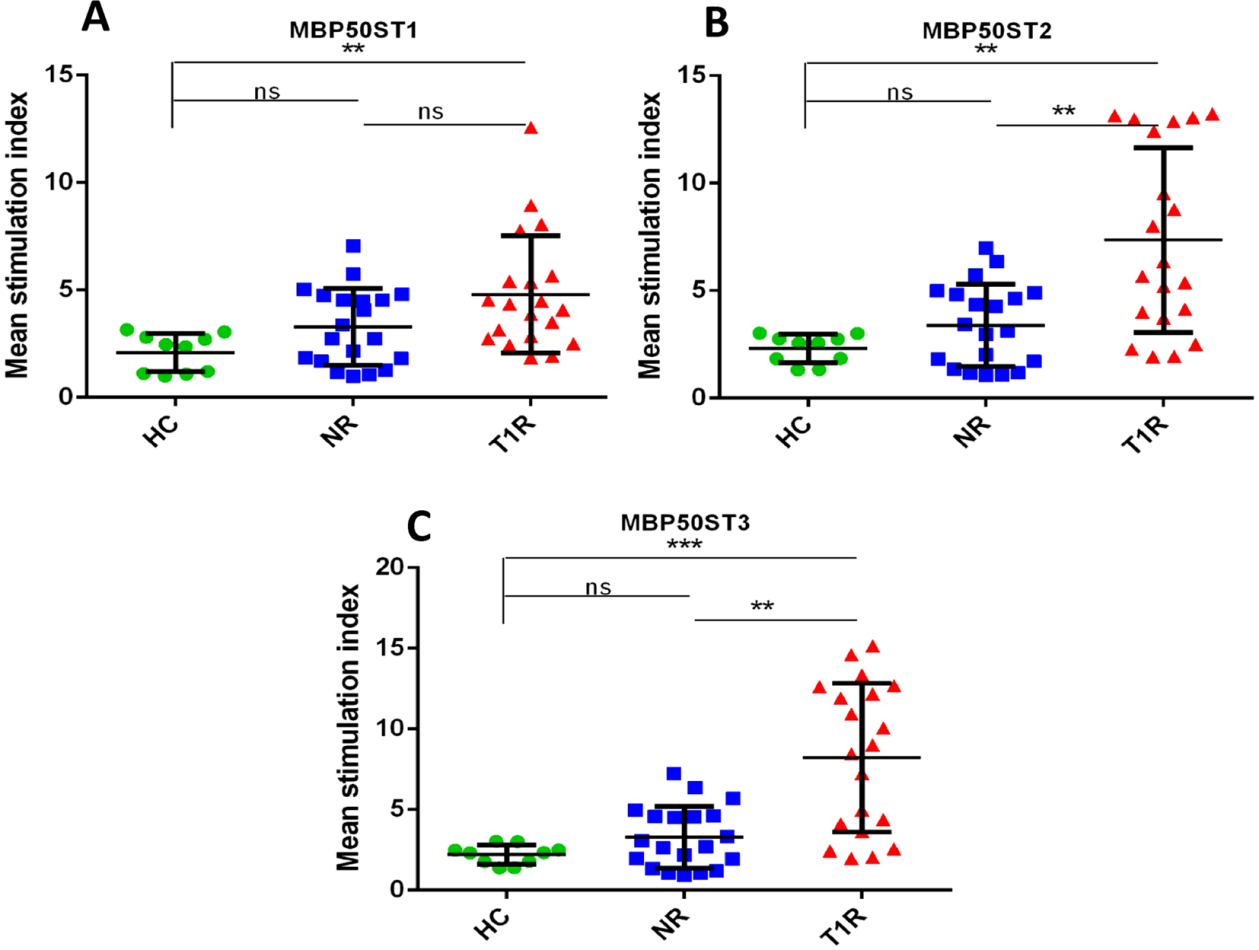
Graph showing mean stimulation index of healthy controls and leprosy patients with mimicking T cell epitopes of MBP with 50S ribosomal protein of *M. leprae* (**A**) MBP50ST1, (**B**) MBP50ST2, (**C**) MBP50ST3. Smooth vertical lines along with horizontal lines represent the mean stimulation index (SI) in each group. Each dot represents the SI of each individual. One-way ANOVA (and non-parametric test) and multiple comparison test (Dunn’s test) were used to find out the difference between SI value obtained in healthy controls and leprosy patients (***p value < 0.001, **p value < 0.01, ns = not significant).

**Figure 7:**
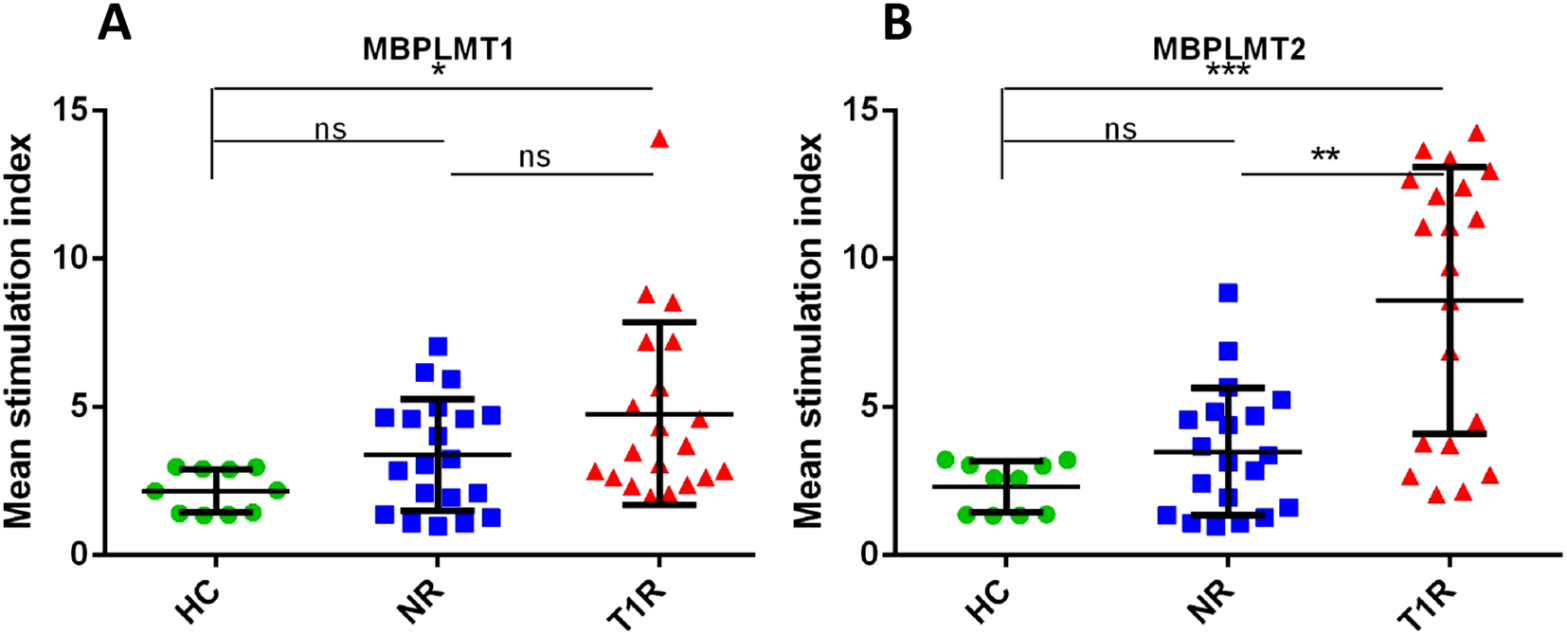
Graph showing mean stimulation index of healthy controls and leprosy patients with mimicking T cell epitopes of MBP with Lysyl tRNA synthetase of *M. leprae* (**A**) MBPLMT1, (**B**) MBPLMT2. Smooth vertical lines along with horizontal lines represent the mean stimulation index (SI) in each group. Each dot represents the SI of each individual. One-way ANOVA (and non-parametric test) and multiple comparison test (Dunn’s test) were used to find out the difference between SI value obtained in healthy controls and leprosy patients (***p value < 0.001, **p value < 0.01, *p value < 0.05, ns = not significant).

**Figure 8:**
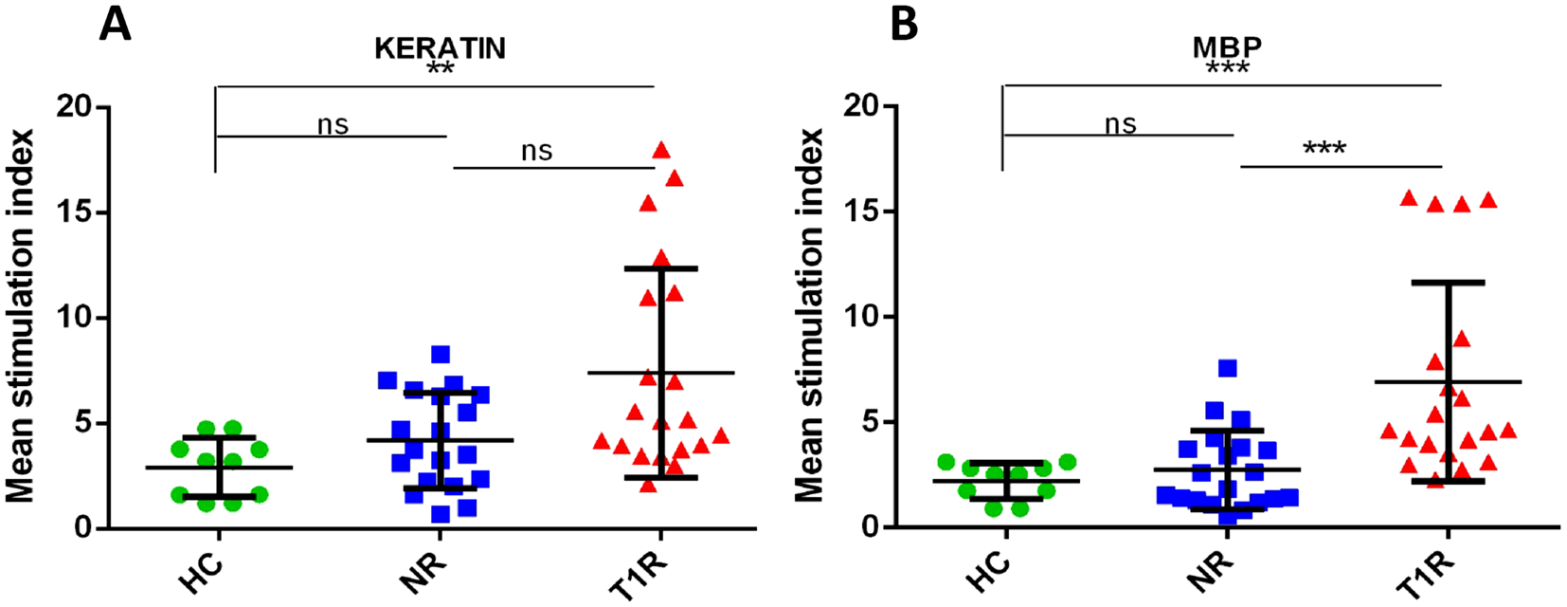
Graph showing mean stimulation index of healthy controls and leprosy patients with host purified protein (**A**) Keratin, (**B**) Myelin basic protein (MBP). Smooth vertical lines along with horizontal lines represent the mean stimulation index (SI) in each group. Each dot represents the SI of each individual. One-way ANOVA (and non-parametric test) and multiple comparison test (Dunn’s test) were used to find out the difference between SI value obtained in healthy controls and leprosy patients (***p value < 0.001, **p value < 0.01, ns = not significant).

Similarly, among three mimicking T cell epitopes of MBP (similar with 50S ribosomal protein of *M. leprae*), we found higher SI in the T1R group with MBP50ST2 and MBP50ST3, when compared to NR (p < 0.01 and p < 0.01, respectively) and HC (p < 0.01 and p < 0.001, respectively). Among two mimicking T cell epitopes of MBP (similar to lysyl tRNA synthetase of *M. leprae*), we found significantly higher SI in the T1R group with MBPLMT2, when compared to NR (p < 0.01) and HC (p < 0.001). We also found higher SI in the T1R group with keratin (Sigma, USA) and MBP (Sigma, USA), when compared to NR (p = ns and p < 0.001, respectively) and HC (p < 0.01 and p < 0.001) (Table 5, Figs. 5, 6, 7 and 8).

**Table 5:** Mean SI values in leprosy patients and healthy controls for each mimicking T cell epitopes.

Sr. no.	Peptide	Healthy controls (n = 10)	Non-reaction n = 20)	Type 1 reaction (n = 20)
1	50ST1 (mean ± SD)	2.439 ± 0.9807	3.224 ± 1.749	4.873 ± 3.69
2	50ST2 (mean ± SD)	2.092 ± 0.7183	3.198 ± 1.667	7.847 ± 4.582
3	LMT1 (mean ± SD)	2.167 ± 0.7137	3.382 ± 2.274	8.148 ± 4.743
4	LMT2 (mean ± SD)	1.954 ± 0.791	3.402 ± 1.883	5.007 ± 3.355
5	MBP50ST1 (mean ± SD)	2.096 ± 0.8875	3.29 ± 1.781	4.799 ± 2.72
6	MBP50ST2 (mean ± SD)	2.304 ± 0.659	3.399 ± 1.912	7.357 ± 4.295
7	MBP50ST3 (mean ± SD)	2.204 ± 0.5986	3.292 ± 1.911	8.216 ± 4.601
8	MBPLMT1 (mean ± SD)	2.172 ± 0.7332	3.392 ± 1.887	4.772 ± 3.07
9	MBPLMT2 (mean ± SD)	2.313 ± 0.8501	3.497 ± 2.159	8.593 ± 4.503
10	MBP (mean ± SD)	2.228 ± 0.8478	2.742 ± 1.861	6.907 ± 4.719
11	KERATIN (mean ± SD)	2.906 ± 1.399	4.195 ± 2.269	7.386 ± 4.971

## Discussion

The association of infectious agents with autoimmune disease has been confirmed by epidemiological, clinical, and experimental findings suggesting existence of cross-reactivity with host ‘self’ antigens and microbial determinants^11–13^.

It has been reported earlier that anti-*M. leprae* monoclonal antibodies cross-react with human nerve and skin components and it has been suggested that this antigenic similarity could also be liable for the event of autoimmune clinical manifestations of leprosy^14, 15^. This antigenic similarity is known as molecular mimicry, it could be linear or conformational.

Heat shock proteins (HSP)s are a phylogenetically conserved family of proteins, which are expressed during chronic inflammation and other forms of physiological stress conditions that lead to both humoral and cellular immune responses against microorganisms^16^. Therefore, HSP may evoke an autoimmune phenomenon in vivo during chronic mycobacterial infections. Earlier, Mycobacterial 65 kDa heat shock protein has been reported to share a carboxy-terminal epitope with human epidermal cytokeratin 1/2^17^. Further, molecular mimicry between cytokeratin-10 of keratin (host) protein and 65 kDa HSP (groEL2) of *M. leprae* was reported and seven B cell epitopes of cytokeratin-10 and HSP 65 were found to be similar^7^. In the present study, we predicted a total of eleven B cell mimicking epitopes of protein HSP65 (*M. leprae*) and protein keratin (host) and evaluated host humoral immune response to determine the antigenic determinant responsible for the autoimmune phenomenon in the T1R group of leprosy. In our observation, the mean level of antibodies against HSP4 and HSP5 were significantly higher in T1R when compared to NR (p < 0.0001, p < 0.001, respectively) and HC (p < 0.001, p < 0.05, respectively). Among all mimicking HSP65 peptides screened, the mean level of antibodies against HSP1 was found to be significantly higher in both T1R and NR when compared to HC (p < 0.0001). The antibody levels against HSP7 was found higher in HC followed by NR (p < 0.01) and T1R (p < 0.0001). The HSPs are well known to express during inflammatory and pathological stress conditions and the occurrence of molecular mimicry at antigen level may induce altered humoral and cell-mediated immune response evoking an autoimmune reaction. The differential level of antibodies against mimicking peptides HSP4, HSP5, and HSP7 among the different groups could be utilized as a predictive biomarker for the development of T1R in leprosy. On the other hand, the epitope HSP1 could be utilized to predict leprosy disease.

High level of antibodies against self-protein, keratin could be an expression of the autoimmune phenomena in leprosy patients, as the majority of leprosy lesions are manifested within the skin, and the occurrence of keratosis is a common phenomenon especially during T1R of tuberculoid leprosy^18, 19^.

*M. leprae* antigens which mimic host antigens may induce an autoimmune reaction against the host’s own antigens, which could explain the immune reaction in tuberculoid leprosy and during a “reversal reaction”, when host tissue is lacking *M. leprae,* whereas, extensive granuloma formation can be seen^14^. Singh et al., have reported that a high level of anti-keratin antibodies (AkAbs) are present in leprosy patients in comparison to healthy controls and the highest level AkAbs was present in T1R followed by LL, BL, ENL, and TT/BT leprosy. It was very interesting to find out that level of AkAbs also clinically correlated with the number of lesions present in leprosy patients^7^. Similarly, in the present study the level of antibodies against three mimicking B cell epitopes of keratin i.e., KER1, KER2, KER4 were found higher when compared to NR (p < 0.0001, p < 0.05 and p < 0.0001, respectively) and HC (p < 0.0001). The antibody responses to these three epitopes were found significantly associated with T1R. Thus, our finding suggests future utilization of these mimicking epitopes as a predictive biomarker for reactions (T1R) in leprosy.

It has been noted that the T1R in BT leprosy patients often occurs suddenly during treatment or after completion of therapy. We still lack data on the exact mechanism of pathogenesis of T1R. Doubts have been raised on the role of mimicking proteins/peptides in the initiation of the reaction. It has been shown earlier that a high level of antibodies against keratin is associated with T1R patients^7^. In the present study we predicted mimicking B cell epitopes and showed a high level of antibodies against a few of these mimicking B cell epitopes. We propose that molecular mimicry between these epitopes of cytokeratin 10 and HSP 65 may potentially lead to acute inflammation in skin lesions. Skin lesions develop scaling in T1R which might also be caused by an autoimmune reaction against these mimicking peptides.

It has also been reported that this immunological response also includes an autoimmune response to nerve antigens which leads to demyelination^20^. It has been shown by Vardhini et al. that there is a molecular mimicry between mycobacterial proteins (ferredoxin-NADP-reductase and a conserved mycobacterial membrane protein) with myelin P0, a protein that aids in compacting myelin through homotypical interactions^21^. In the above study, it was noted only by using bioinformatics tools however, in the present study we performed wet-lab validation along with using bioinformatics tools. Earlier, it was reported that in LL patients circulating immune complexes (CIC) contain MBP as an antigen. Liberation of MBP after *M. leprae* nerve damage may elicit anti-MBP antibodies, which react with peripheral nerve MBP. This mechanism may be responsible for demyelination and destruction of nerves in leprosy^22^. Earlier, it was also reported that MBP antibodies are associated directly or indirectly with neuro-degeneration in leprosy patients^20, 23^.

In the present study among four B cell mimicking epitopes of protein 50S ribosomal protein, lysyl tRNA synthetase (*M. leprae*), and myelin basic protein (host), we found a higher level of antibodies against MBP50SB1 in T1R and a lower level against MBP50SB2 when compared to NR (ns and p < 0.01, respectively). Similarly, we observed a higher level of antibodies in leprosy patients when compared to HC for MBPLMB1 and MBPLMB2. We also found a lower level of antibodies in T1R for MBPLMB1 (p < 0.05) and a higher level for MBPLMB2 (p < 0.01) when compared to NR. MBPLMB2 was found to be significantly associated with T1R. Singh et al., reported a high level of anti-MBP antibodies in leprosy patients across the spectrum and also found that four B cell epitopes of myelin A1 and *M. leprae* proteins, 50S ribosomal L2, and lysyl tRNA synthetase were cross reactive^8^. Earlier reports have shown that *M. leprae* binds to a nerve protein, the myelin protein P zero (P0) which is specific to the peripheral nerve and may be important in the initial step of *M. leprae* binding and invasion of Schwann cells. For the first time, our data indicate that these mimicking B cell epitopes might be responsible for pathological destruction of myelin sheath which may lead to nerve damage and cause deformities and permanent loss of nerve function in leprosy patients.

On the evaluation of lymphocyte proliferation activity against mimicking T cell epitopes we observed that two mimicking epitopes i.e., 50ST2 and LMT1 of *M. leprae* proteins, 50S ribosomal protein, and Lysyl tRNA synthetase, were found significantly associated with the T1R group when compared to NR (p < 0.01 and p < 0.01, respectively) and HC (p < 0.001 and p < 0.01, respectively).

Similarly, two mimicking epitopes i.e., MBP50ST2 and MBP50ST3 of host protein MBP and *M. leprae* protein 50S ribosomal protein, significantly stimulated cells in the T1R group when compared to NR (p < 0.01 and p < 0.01, respectively) and HC (p < 0.01 and p < 0.001, respectively). Further, one mimicking epitope of MBP i.e., MBPLMT2, and *M. leprae* protein Lysyl tRNA synthetase, significantly stimulated cells in the T1R group when compared to NR (p < 0.01) and HC (p < 0.001) group. The purified host proteins Keratin and MBP were also evaluated for reference purposes and we observed that higher SI is associated with the T1R group when compared to NR (p = ns and p < 0.001, respectively) and HC (p < 0.01 and p < 0.001) group.

The T cell epitopes were found to be associated with high CMI for keratin and MBP in the T1R group of leprosy patients. We observed earlier that auto-reaction can be induced in experimental animals after hyper immunization with *M. leprae* sonicated antigens and this induced auto-reaction is transferrable to naïve mice by immune cells^7, 8^. The higher SI by mimicking T cell epitopes suggests high cell mediated immune response against mimicking T cell epitopes and for the first time showed the involvement of mimicking epitopes associated autoimmune reaction in the pathogenesis of reaction as DTH induced by self-antigens.

This study indicated that the possible key factors responsible for the high autoantibodies against keratin and MBP observed earlier by Singh et al*.*^7^ and Singh et al*.*^8^ are mimicking B cell epitopes between host protein/s and M. leprae protein/s. Further, high levels of antibody response by the host during T1R to the mimicking epitopes of *M. leprae* might strongly indicate that antibody-dependent cell-mediated cytotoxicity (ADCC) might be also involved in tissue damage in type 1 reactions in leprosy.

Hence, we conclude from our study that these mimicking B and T cell epitopes are responsible for auto-reaction observed in the T1R group. These mimicking B and T cell epitopes might be responsible for acute inflammation of skin lesions or nerves or both.

## Conclusion

This study indicated that mimicking host and *M. leprae* specific B cell and T cell epitopes could be utilized for the development of a serological test for diagnosis of Type 1 reaction. Early identification of a case of leprosy with type 1 reaction may facilitate early diagnosis and treatment before the onset of deformity consequent to nerve damage in T1R leprosy. Further, this study also indicated that T cells might also be involved in ADCC to explain the mechanism of tissue damage. However, to establish such a mechanism of T cell cytotoxicity further studies have to be carried out to explain the tissue damage in T1R.
